# Admission Hyperglycemia and Acute Myocardial Infarction: Outcomes and Potential Therapies for Diabetics and Nondiabetics

**DOI:** 10.1155/2012/704314

**Published:** 2012-07-09

**Authors:** Anjan K. Chakrabarti, Priyamvada Singh, Lakshmi Gopalakrishnan, Varun Kumar, Meagan Elizabeth Doherty, Cassandra Abueg, Weici Wang, C. Michael Gibson

**Affiliations:** ^1^Cardiovascular Division, Department of Medicine, Beth Israel Deaconess Medical Center, Harvard Medical School, Boston, MA 02215, USA; ^2^PERFUSE Angiographic Core Laboratories and Data Coordinating Center, Beth Israel Deaconess Medical Center, Boston, MA 02215, USA

## Abstract

Hyperglycemia, in both diabetic and nondiabetic patients, has a significant negative impact on the morbidity and mortality of patients presenting with an acute myocardial infarction (AMI). Contemporary evidence indicates that persistent hyperglycemia after initial hospital admission continues to exert negative effects on AMI patients. There have been a number of studies demonstrating the benefit of tight glucose control in patients presenting with AMI, but a lack of convincing clinical data has led to loose guidelines and poor implementation of glucose targets for this group of patients. The CREATE-ECLA study, which hypothesized that a fixed high dose of glucose, insulin, and potassium (GIK) would change myocardial substrate utilization from free fatty acids to glucose and therefore protect ischemic myocardium, failed to demonstrate improved clinical outcomes in AMI patients. Studies that specifically investigated intensive insulin therapy, including DIGAMI-2 and HI-5, also failed to improve clinical outcomes such as mortality. There are a number of reasons that these trials may have fallen short, including the inability to reach glucose targets and inadequate power. There is now a need for a large placebo-controlled randomized trial with an adequate sample size and adherence to glucose targets in order to establish the benefit of treating hyperglycemia in patients presenting with AMI.

## 1. Background

Major advances in cardiovascular disease, and specifically the treatment of acute coronary syndrome (ACS), have had a significant impact on the morbidity and mortality of patients with acute myocardial infarctions (AMI). Despite these advances, diabetes continues to put patients with and without a prior history of myocardial infarction at significant cardiovascular risk [1].  The presence of diabetes doubled the age-adjusted risk for cardiovascular disease in men and tripled it in women in the Framingham Heart Study, and it remained an independent risk factor even after adjusting for  age,  hypertension,  smoking,  hyperlipidemia,  and  left ventricular hypertrophy [2]. Furthermore, there is a graded rise in cardiovascular risk with increasing hyperglycemia in patients with overt diabetes. In fact, as demonstrated by a meta-analysis of 13 prospective cohort studies, for every one-percentage point increase in glycosylated hemoglobin (HbA1c), the relative risk for any cardiovascular event was 1.18 (95% CI 1.10–1.26) [3]. 

It has been well documented that with adequate glycemic control, cardiovascular outcomes improve in patients with both type 1 and type 2 diabetes [4]. Interestingly, intensive glycemic control does not have the same effect, particularly in long-standing type 2 diabetics. In the ACCORD study, 10,250 patients with long-standing type 2 diabetes were randomly assigned to either intensive or standard glycemic control. After a median followup of 3.7 years, intensive therapy was discontinued due to a higher number of total and cardiovascular deaths in subjects assigned to intensive therapy (median HbA1C 6.4 percent) compared with the standard treatment group (median HbA1C 7.5 percent) [5].

This raises the question: what are the effects of hyperglycemia, standard glycemic control, and intensive glycemic control in the setting of acute myocardial infarction and/or acute coronary syndrome? How do these effects differ in long-standing diabetic and nondiabetic populations? While there has been extensive study in the area of hyperglycemia and cardiovascular outcomes in AMI, there is no consensus regarding blood glucose targets and the benefits of treating hyperglycemia in this setting. This in turn is leading to poor control of blood glucose in the AMI setting, an area where further studies could yield significant improvement in cardiovascular outcomes.

## 2. The Prognostic Role of Glucose Values in Acute Myocardial Infarction

**Figure 1:**
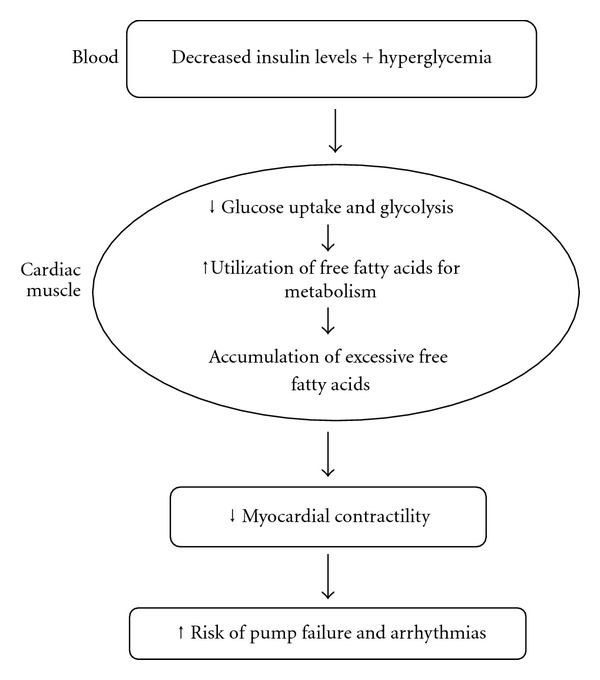
Hyperglycemia and its effect on cardiac function.

The prevalence of admission hyperglycemia (glucose levels of >140 mg/dL) in different epidemiological studies ranges from 51% to >58% of patients admitted with AMI [6]. In patients with AMI, hyperglycemia at the time of admission regardless of diabetic status has been tied to both long- and short-term negative outcomes [7, 8]. A number of contemporary investigators, however, have challenged this notion and demonstrated that hyperglycemia after hospital admission may yield a more important prognostic role than admission hyperglycemia in terms of morbidity and mortality [9, 10]. Suleiman and colleagues, for instance, were able to demonstrate that fasting glucose was superior to admission glucose in predicting 30-day mortality in 735 nondiabetic AMI patients [11]. Loomba and Arora performed an extensive systemic review and were able to demonstrate that persistent glucose levels offer a better model to predict ACS mortality than on-admission glucose levels [12]. It has been demonstrated that the use of insulin to lower glucose concentrations decreased negative outcomes in patients with hyperglycemia and myocardial infarction (see Section 3); however, a mechanism by which hyperglycemia may be a causal factor in poor outcomes in AMI remains a topic of debate. It has been proposed that in AMI patients, decreased levels of blood insulin associated with hyperglycemia may lead to a decrease of glycolytic substrate for cardiac muscle. As a result, the heart has to depend on alternate substrates such as free fatty acids for its metabolism. The accumulation of excessive free fatty acids results in the reduction of myocardial contractility and increases the risk of pump failure and arrhythmias (Figure 1).  This challenges the assumption that hyperglycemia is simply a “marker” of the stress response mediated by cortisol and noradrenaline [13]. A meta-analysis by Capes and colleagues in 2000 supported this hypothesis by demonstrating that among nondiabetic patients, those with glucose concentrations between 110 and 143 mg/dL had a 3.9-fold higher risk of death and that those with glucose values between 144 and 180 mg/dL had a 3-fold higher risk of heart failure or cardiogenic shock. Similarly, diabetics with glucose concentrations between 180 and 196 mg/dL had an increased risk of death (relative risk 1.7) [7]. 

In addition to decreased contractility, pump failure, and arrhythmia, hyperglycemia in AMI may affect coronary perfusion prior to and following percutaneous coronary intervention (PCI). A 2005 observational analysis by Timmer and colleagues sought to determine how hyperglycemia affected coronary perfusion prior to revascularization in ST-segment elevation myocardial infarction (STEMI) [14]. In 460 consecutive patients with STEMI who were treated with PCI, 70% had serum glucose levels  ≥140 mg/dL (7.8 mmol/L) on admission, but only 14 percent had a history of diabetes. They were able to demonstrate that the patients with hyperglycemia were significantly less likely to have TIMI grade  3  (normal) flow prior to PCI compared to those with normoglycemia (Table 1). This finding complements those by Lazerri and colleagues in 2010, who were able to demonstrate that glucose serum levels measured after mechanical revascularization were independent predictors of in-hospital mortality in STEMI patients without previously-known diabetes [15]. Indeed, acute hyperglycemia has been associated with increased platelet activation in diabetic and nondiabetic patients [16]; coupled with evidence that acute hyperglycemia increases inflammatory responses during STEMI [17, 18], these findings could explain an impairment in coronary flow that reflects a prothrombotic state and/or endothelial dysfunction associated with hyperglycemia, leading to a greater stress response.

Another interesting aspect of the prognostic value of hyperglycemia in AMI is the differences observed in nondiabetic and diabetic patients. In their 2011 study, Lazerri and colleagues observed that in a cohort of STEMI patients undergoing mechanical revascularization, in-hospital peak glycemia was an independent predictor for early death in patients without previously known diabetes, but not in diabetic STEMI patients. At followup, in hospital peak glycemia was able to affect long-term survival in both diabetic and nondiabetic patients. This data underscores the clinical importance of the prognostic role of in-hospital glucose values and strongly suggests that different glucose targets and thresholds may be pursued in diabetic and nondiabetic STEMI patients [19]. 

## 3. Glycemic Control in Patients with Acute Myocardial Infarction

Controlling hyperglycemia during AMI admissions has been the target of a great deal of basic and clinical research, with results primarily trending toward a benefit. The optimal “glucose target” has been elusive, and contemporary guidelines reflect this [20, 21].  A  prospective study of 32 patients demonstrated that insulin infusion reduced inflammatory and clotting mediators in the plasma, with a concomitant reduction in enzymatic infarct size in subjects with STEMI receiving fibrinolytics [22]. Recent experimental data also supports the beneficial effects of insulin infusion among patients with AMI. Wong and colleagues were able to demonstrate that insulin started 5 minutes prior to reperfusion in white rabbits significantly reduced infarct size following regional ischemia and reperfusion in a dose-dependent manner [23]. 

Although this data looks promising, previous large prospective studies of insulin in AMI have resulted in varying levels of benefit. Many of these studies involved infusion of a fixed high dose of glucose, insulin, and potassium (GIK) to change myocardial substrate utilization from free fatty acids to glucose. It was hypothesized that this, in turn, would protect the ischemic myocardium [24, 25]. In the CREATE-ECLA study, which enrolled 20,000 patients, this GIK regimen did not demonstrate improved outcomes in AMI [26]. In this trial, 40% of the total subjects became hyperglycemic (>144 mg/dL) in the postrandomization period; of these, the majority (62%) were in the GIK group. Subjects with a postrandomization glucose of >144 mg/dL had a 2.5-fold higher risk of mortality compared to subjects with a glucose <126 mg/dL. While GIK was associated with a significant reduction in mortality and heart failure at 30 days after MI, the overall effect was neutralized by an increase in mortality and heart failure with this regimen in the immediate 3-day post-MI period. This initial increase in poor outcomes was likely mediated by the fact that GIK infusion frequently induced hyperglycemia and volume overload, which may have mitigated the beneficial effects of insulin in the regimen.

**Figure 2:**
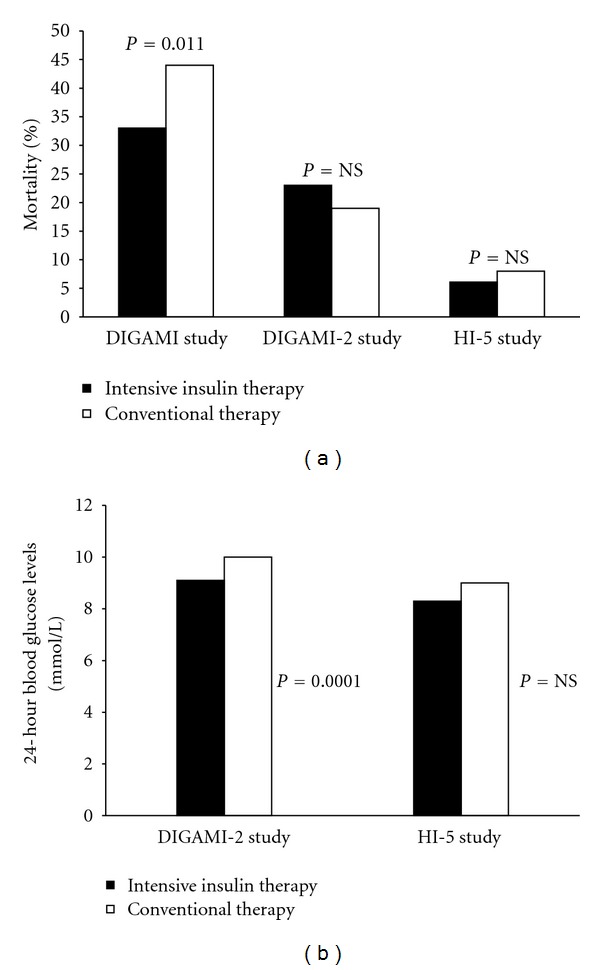
(a) Mortality benefit of intensive insulin therapy over conventional therapy in clinical trials. (b) Reduction in 24 hour blood glucose levels with intensive insulin therapy over conventional therapy in clinical trials.

Apart from GIK regimens, there have been other studies that have infused insulin in hyperglycemic patients with AMI with the intent to reduce glucose, including the DIGAMI, DIGAMI-2 and HI-5 trial. While the DIGAMI study was able to demonstrate a decrease in both mortality and mean 24-hour blood glucose levels, the DIGAMI-2 and HI-5 studies showed no such significant decrease (Figure 2) [27–29]. Importantly, the varying glucose target levels of these studies make it difficult to compare their results and infer a firm conclusion. Of note, blood glucose levels in these trials were not adequately lowered, the trials were underpowered to show an effect on mortality, and the infusions were started more than 6 hours after symptom onset, when it may have been too late to salvage the myocardium. Despite these shortcomings, insulin infusion was shown to lower the risk of heart failure (absolute risk reduction of 10%) and reinfarction (absolute risk reduction of 3.4%) [27, 29]. 

## 4. Future Directions and Areas of Further Study

The benefit of insulin infusion in patients with hyperglycemia in ACS has yet to be demonstrated convincingly in a large prospective clinical trial. While there is significant evidence that admission and postadmission hyperglycemia is associated with increased mortality and morbidity in AMI, there is no consensus regarding the targets and the benefits of treating hyperglycemia in this setting. This is illustrated by the 2009 ACC/AHA focused update on STEMI, which makes a rather weak recommendation (Table 2) for the use of an insulin-based regimen to achieve and maintain blood glucose levels below 180 mg/dL (10 mmol/L), consistent with the 2007 update by Antman and colleagues (level of evidence B) [20, 21]. It should be noted this recommendation does not discriminate based on prior diabetes history. Similarly, Kosiborod and colleagues, who performed an extensive review on the topic of hyperglycemia and AMI, have recommended glucose treatment targets within the “conservative range” of 140–180 mg/dl [30]. This falls within the range demonstrated to be beneficial by Lazerri and colleagues, who in 2010 demonstrated in 252 nondiabetic STEMI patients undergoing mechanical revascularization, that peak glycemia >180 mg/dL was associated with the elevated mortality, whereas patients with peak glycemia comprised between 140 and 180 mg/dL exhibited attenuated mortality rates [19]. 

If the lowering of glucose levels with insulin infusion can demonstrate a reduction in adverse cardiac outcomes over and above the benefits provided by reperfusion therapy, it would be a significant improvement in the management of ACS in the reperfusion era. Indeed, this was reflected in a 2008 AHA statement on hyperglycemia in ACS, where the guidelines emphasized the need for a large prospective randomized study examining this issue. Specifically, it recommends randomized multicenter trials which should include hyperglycemic patients both with and without preexisting diabetes (suggested definition is plasma glucose >140 mg/dL at admission), should use safe and effective protocols for glucose control, and should afford sufficient statistical power to assess mortality as a primary outcome [31]. 

## 5. Conclusion

While significant advances have been made in the treatment and prevention of cardiovascular disease, hyperglycemia in the setting of acute myocardial infarction remains an area where aggressive therapy could lead to a significant improvement in outcomes. Current guidelines stipulate a blood glucose goal of 180 mg/dL with the use of an insulin-based regimen in the setting of AMI; however, a more aggressive target has not been universally recommended primarily due to lack of convincing evidence. While observational and small prospective studies, as well as experimental data, have demonstrated a benefit in driving down a glucose target using insulin infusion [12], a randomized trial with an adequate sample size and stringent adherence to glucose goals is now necessary.

## Figures and Tables

**Table 1:** Initial TIMI flow grade according to admission glucose. Worse initial Thrombolysis in Myocardial Infarction (TIMI) flow grade is demonstrated in those with hyperglycemia.

	Glucose < 7.8 mmol/L (<140 mg/dL)	Glucose ≥7.8 mmol/L (≥140 mg/dL)	*P* value
TIMI flow grade 3	38 (28%)	38 (12%)	<0.001^∗^
TIMI flow grade 2	32 (23%)	65 (20%)	
TIMI flow grade 1	7 (5%)	42 (13%)	
TIMI flow grade 0	61 (44%)	177 (55%)	0.03^†^

^∗^As compared to TIMI flow grade 2 to 0.

^†^As compared to TIMI flow grade 1 to 3.

Table from [14].

**Table 2:** Recommendations for intensive glucose control in STEMI.

2004/2005/2007 recommendations: 2004 STEMI guidelines	2009 joint STEMI/PCI focused update recommendations	Comments
Class I		

(1) An insulin infusion to normalize blood glucose is recommended for patients with STEMI and complicated courses (*level of evidence: B*).		Recommendation is no longer current. See 2009 Class IIa recommendation no. 1.

Class IIa		

	(1) It is reasonable to use an insulin-based regimen to achieve and maintain glucose levels less than 180 mg/dL while avoiding hypoglycemia∗ for patients with STEMI with either a complicated or uncomplicated course (16, 94–96) (*level of evidence: B*).	New recommendation
(1) During the acute phase (first 24 to 48 hours) of the management of STEMI in patients with hyperglycemia, it is reasonable to administer an insulin infusion to normalize blood glucose even in patients with an uncomplicated course (*level of evidence: B*).		Recommendation is no longer current. See 2009 Class IIa recommendation no. 1.

^∗^There is uncertainty about the ideal target range for glixose necessary to achieve an optimal risk-benefit ratio.

Recommendations for Intensive Glucose Control in STEMI.

Table from [20].
